# Updated Guidelines on Managing Drug Interactions in the Treatment of HIV-Related Tuberculosis

**Published:** 2014-03-28

Guidelines for managing pharmacologic interactions that can result when patients receive antiretroviral drugs for treatment of human immunodeficiency virus (HIV) infection together with rifamycin antibiotics for treatment of tuberculosis (TB) have been published previously (1–4). Newly updated guidelines, developed by CDC in collaboration with experts from other key national and international institutions, are now available at http://www.cdc.gov/tb/publications/guidelines/tb_hiv_drugs/default.htm.

The updated guidelines include recommendations for use of newer antiretroviral drugs, including those in new classes, such as CCR5 receptor antagonists and integrase inhibitors. The new guidelines provide additional recommendations regarding use of rifampin with antiretroviral therapy; these recommendations are critical in regions where rifabutin is unavailable. New features of the guidelines include 1) summaries of clinical experience with use of specific antiretroviral regimens during TB treatment (in addition to pharmacokinetic data), 2) a table summarizing clinical experience with key antiretroviral regimens and providing recommended regimens, and 3) sections on treatment for special populations, including persons with latent TB infection, young children, pregnant women, and patients with drug-resistant TB. The online guidelines will be updated as necessary to provide clinicians with the latest information.
